# Supplementary material for: Population Structure and Resource Dynamics of Three Schizothoracinae Species in the Duoxiong Zangbo River Tributary of the Yarlung Zangbo River, Tibet: Threat Assessment and Conservation Insights
Source: Animals (Basel). 2025 Aug 10;15(16):2340. doi: 10.3390/ani15162340 (PMC12383017; doi:10.3390/ani15162340)
Supplement: Supplementary file 1 [file animals-15-02340-s001.zip › animals-3772999-supplementary.pdf]

Table S1. Catch data from sampling sites

Species name		Abundances(ind.)	CPUE(g/h)
O. stewarti	P1	9	265.5508
	P2	8	131.0692
	P3	3	118.5983
	P4	7	287.1433
	P5	15	638.845
	P6	3	153.7917
P. dipogon	P1	5	115.4042
	P2	8	327.2708
	P3	11	269.5525
	P4	22	834.3042
	P5	44	1435.022
	P6	7	250.9792
S. o'connori	P1	12	298.8017
	P2	1	33.00833
	P3	14	233.405
	P4	4	243.1192
	P5	5	277.7967
	P6	15	764.7633
